# Design, Synthesis of *N*-phenethyl Cinnamide Derivatives and Their Biological Activities for the Treatment of Alzheimer’s Disease: Antioxidant, Beta-amyloid Disaggregating and Rescue Effects on Memory Loss

**DOI:** 10.3390/molecules23102663

**Published:** 2018-10-16

**Authors:** Tian Chai, Xiao-Bo Zhao, Wei-Feng Wang, Yin Qiang, Xiao-Yun Zhang, Jun-Li Yang

**Affiliations:** 1 School of Pharmacy, Lanzhou University, Lanzhou 730000, China; chait16@lzu.edu.cn (T.C.); qiangy@lzu.edu.cn (Y.Q.); 2 CAS Key Laboratory of Chemistry of Northwestern Plant Resources and Key Laboratory for Natural Medicine of Gansu Province, Lanzhou Institute of Chemical Physics (LICP), Chinese Academy of Sciences (CAS), Lanzhou 730000, China; zhaoxb08@licp.cas.cn (X.-B.Z.); wwf2004@126.com (W.-F.W.)

**Keywords:** Alzheimer’s disease, gx-50, DPPH assay, ThT assay, transgenic *Drosophila* model assay

## Abstract

Gx-50 is a bioactive compound for the treatment of Alzheimer’s disease (AD) found in Sichuan pepper (*Zanthoxylum bungeanum*). In order to find a stronger anti-AD lead compound, 20 gx-50 (**1**–**20**) analogs have been designed and synthesized, and their molecular structures were determined based on nuclear magnetic resonance (NMR) and mass spectrometry (MS) analysis, as well as comparison with literature data. Compounds **1**–**20** were evaluated for their anti-AD potential by using DPPH radical scavenging assay for considering their anti-oxidant activity, thioflavin T (ThT) fluorescence assay for considering the inhibitory or disaggregate potency of A*β*, and transgenic *Drosophila* model assay for evaluating their rescue effect on memory loss. Finally, compound **13** was determined as a promising anti-AD candidate.

## 1. Introduction

Alzheimer’s disease (AD) is a neurodegenerative disorder that mainly occurs in the elderly. It is characterized by intelligence decline and memory loss, as well as changes in emotions and personality [1]. There are approximately 36 million AD patients worldwide, and this figure will double every 20 years [2]. Since the 1990s, a great deal of financial support has been invested to explore the molecular pathogenesis of AD, which had provided robust support to develop effective pharmacological treatments [3]. Currently, the clinically used AD drugs mainly include cholinesterase inhibitors, including tacrine, donepezil, rivastigmine, galanthamine, and huperzine A, and the N-methyl-D-aspartate (NMDA) receptor antagonist, that is memantine. These drugs could alleviate cognitive symptoms in this disease. Unfortunately, there is no effective means to cure, or only slow, the progression of this disease [4,5].

The pathogenesis of AD has not yet been fully understood. Therefore, a variety of AD hypotheses have been proposed, such as the amyloid cascade hypothesis, the Tau phosphorylation hypothesis, the neurovascular hypothesis, the oxidative stress hypothesis, and the immune hypothesis [4]. One of the major pathological hallmarks of AD is the aggregation of amyloid plaques in the brain. Its essence are the fibrils of the amyloid-β peptide (Aβ) [6,7]. Oxidative stress also plays an important role in the development and progress of AD, and 1,1-diphenyl-2-picrylhydrazyl (DPPH) assay is one of the most common methods to evaluate the anti-oxidative activity of compounds [8,9]. Moreover, transgenic *Drosophila* that can express AD-related proteins has been successfully used to screen the potential anti-AD compounds [10].

*N*-[2-(3,4-Dimethoxyphenyl)ethyl]-3-phenyl-acrylamide (gx-50), a potential drug candidate for the treatment of AD, was isolated from Sichuan pepper (*Zanthoxylum bungeanum*). Gx-50 could penetrate the blood brain barrier (BBB) and enter the brain tissue to improve the cognitive function of dementia mice, and meanwhile could reduce the A*β* plaques in brain tissues [11,12,13]. In order to find a much stronger drug candidate for the treatment of AD, 20 analogues of gx-50 were designed and synthesized in this study. Consequently, the DPPH assay and thioflavin T (ThT) assay were employed to evaluate their anti-oxidative activity and their inhibition and disaggregation on Aβ aggregation. Finally, Pavlov’s olfactory memory test was used to evaluate their rescue effects on memory loss as analyzed on the behavior of AD model flies. Herein, we will report the design, synthesis, and anti-AD bioactivity of these gx-50 derivatives.

## 2. Results and Discussion

### 2.1. Chemistry

**Scheme 1:**
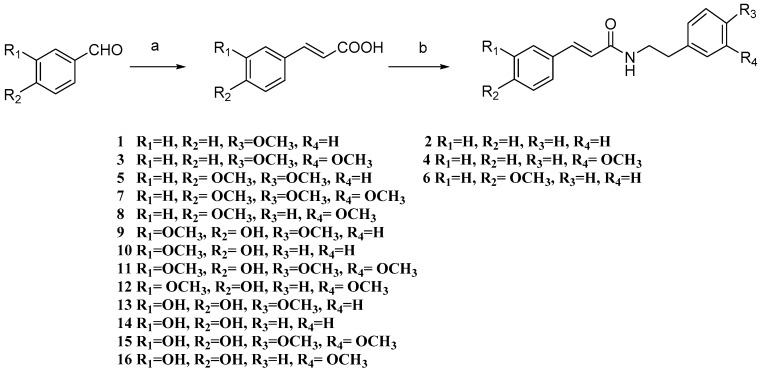
Synthesis of compounds **1**–**16**. Reagents and conditions: (**a**) anhydrous pyridine, piperidine, malonic acid, oil bath, 90 °C, 4 h; (**b**) cinnamic acid, amine, EDCI, DMAP, DMF, room temperature, overnight.

**Scheme 2:**
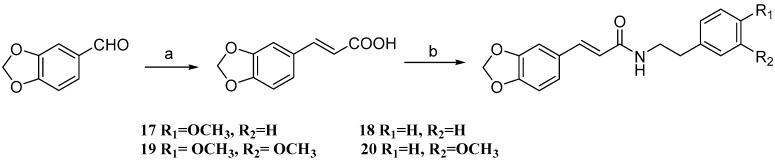
Synthesis of compounds **17**–**20**. Reagents and conditions: (**a**) anhydrous pyridine, piperidine, malonic acid, oil bath, 90 °C, 4 h; (**b**) cinnamic acid, amine, EDCI, DMAP, DMF, room temperature, overnight.

The synthesis of the gx-50 derivatives is described in Scheme 1 and Scheme 2. All compounds were synthesized by following previously reported methods with little modifications [14]. Specifically, the cinnamic acid was synthesized firstly by the substituted aromatic aldehyde with malonic acid through the Knoevenagel reaction. Then in the presence of 3-(ethyliminomethylideneamino)-*N*,*N*-dimethylpropan-1-amine hydrochloride (EDCI) and 4-dimethylaminopyridine (DMAP), the cinnamic acids with different substitutions reacted with 4-methoxyphenethylamine, 2-phenylethylamine, 3,4-dimethoxyphenethylamine, and 3-methoxyphenethylamine, respectively. Finally, one molecule of water was removed at room temperature to produce the corresponding target compounds **1**–**20**. Among them, compounds **12**, **16**, and **20** have never been reported by searching the SciFinder database. Regarding the known compounds, their chemical structures were determined and confirmed by NMR and MS data analysis as well as literature comparison. Some of them were reported to show potent biological activities. For example, compounds **1**, **9**, and **13** exhibited indution of apoptosis in U-937 cells at 100 μM [15]. Compound **3** and **11** had an antihyperglycemic effect as inhibition percentage of 20.1% and 30.7% in sucrose-loaded model (SLM) at a dosage of 100 mg/kg-body weight [16,17]. Compound **5** inhibited platelet aggregation with the IC_50_ value of 2.6 μM [18]. Compound **10** and **11** showed anti-inflammatory activity with 50% NO inhibition concentration as 14.08 μM and 15.08 μM, respectively [19,20]. Compound **10** and **14** revealed 5-lipoxygenase inhibitory activity with the IC_50_ value of 0.12 μM and 1 μM, respectively [21]. Compound **15** could inhibit tyrosinase as the IC_50_ value of 0.6 mM [22,23]. In this study, all compounds **1**–**20** were evaluated for their anti-AD bioactivity via DPPH assay, ThT test, and Pavlov’s olfactory memory test for the first time.

### 2.2. Pharmacology

#### 2.2.1. Determination of Anti-Oxidant Activity Based on DPPH Assay

The oxidative damage to neurons is closely related to the pathogenesis of AD [24]. In this article, the anti-oxidative activity of compounds **1**–**20** was evaluated by DPPH assay with Vitamin C (Vc) as the positive control [25]. The free radical scavenging ability was evaluated based on the UV absorbance change of the solution measured at 517 nm using a microplate reader [26]. As shown in Table 1, the compound concentration and their anti-oxidative activity exhibited a favorable concentration-dependent relationship. In order to discuss their structure-activity relationship, compounds **1**–**20** were classified into three groups as follows. The first group contains compounds **1**–**8**, which only contained methoxy substituents. These compounds showed weak DPPH scavenging activity. The second group contains compounds **9**–**16**, which had the substituents of hydroxyls and methoxyls. These compounds exhibited comparable DPPH scavenging activity, compared to the positive control. Moreover, compounds **13**–**16** with two hydroxyls showed much stronger activity than compounds **9**–**12** with just one hydroxyl substituent. The third group contains compounds **17**–**20**, which contained 3,4-(methylenedioxy)cinnamic acid moiety. These compounds showed the weakest DPPH scavenging activity. In addition, the DPPH scavenging rate of compound **3** (gx-50) was less than 10%, and its derivatives **9**–**16** exhibited stronger DPPH scavenging ability ranging from 16.69 ± 0.46 to 60.85 ± 0.37. As discussed above, the phenolic hydroxyl may be a functional group for the anti-oxidant ability of gx-50 derivatives. This summarized structure-activity relationship is consistent with the previously reported conclusion [27].

#### 2.2.2. Inhibition of Cu^2+^-Induced Aβ Aggregation and Disaggregation

The inhibitory activities of compounds **1**–**20** on copper-mediated Aβ_1–42_ aggregation and disaggregation were evaluated by using ThT assay [28,29] with resveratrol and curcumin as positive controls. As shown in Table 2, the inhibitory and disaggregate potency of compounds **1**–**4** were less than 5%. Specifically, the inhibitory and disaggregate potency of compound **3** (gx-50) were 1.73 ± 2.15% and 2.39 ± 1.35%, respectively. However, when R_2_ becomes methoxyl group, as in compounds **5**–**8**, the inhibitory and disaggregate potency obviously increased except for compound **8**. Therefore, R_2_ may be an active position that could increase inhibitory or disaggregate potency of the test compounds. The inhibitory and disaggregate potency of compounds **9**–**12** were less than 10%. Compounds **13**–**16** exhibited equal or better inhibitory and disaggregate potency than curcumin and resveratrol. Compounds **17**–**20** had little inhibitory or disaggregate potency. The most active compound was compound **15**, with 61.85 ± 1.70% and 64.44 ± 0.76%, respectively. As discussed, the catechol part could be the bioactive part for the gx-50 derivatives to inhibit Aβ aggregation and disaggregate Aβ aggregate.

#### 2.2.3. Pavlovian Olfactory Aversive Immediate Memory Test

**Figure 1:**
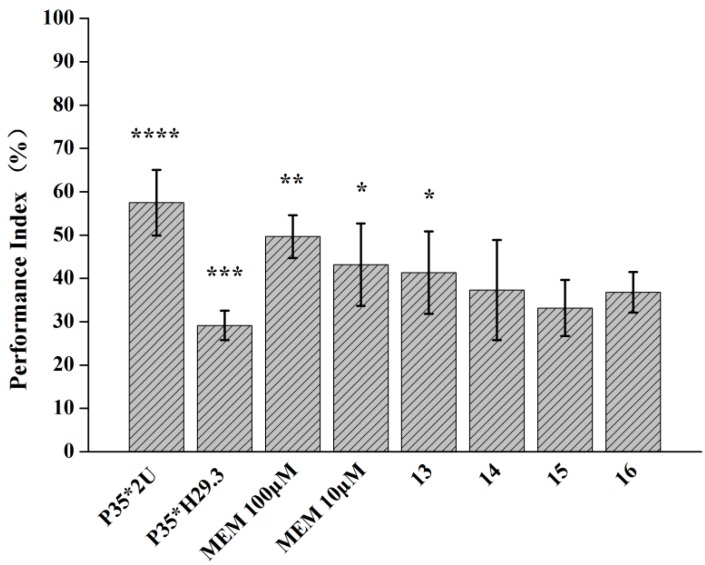
Differential effects of compound **3** and compounds **13**–**16** on rescuing memory deficit on a *Drosophila* Alzheimer’s disease (AD) model at 10 μM. The data are shown as means ± SD and analyzed by one-way ANOVA; * *p* <0.05; ** *p* < 0.01; *** *p* < 0.001; *n* ≥ 20. MEM = memantine.

**Figure 2:**
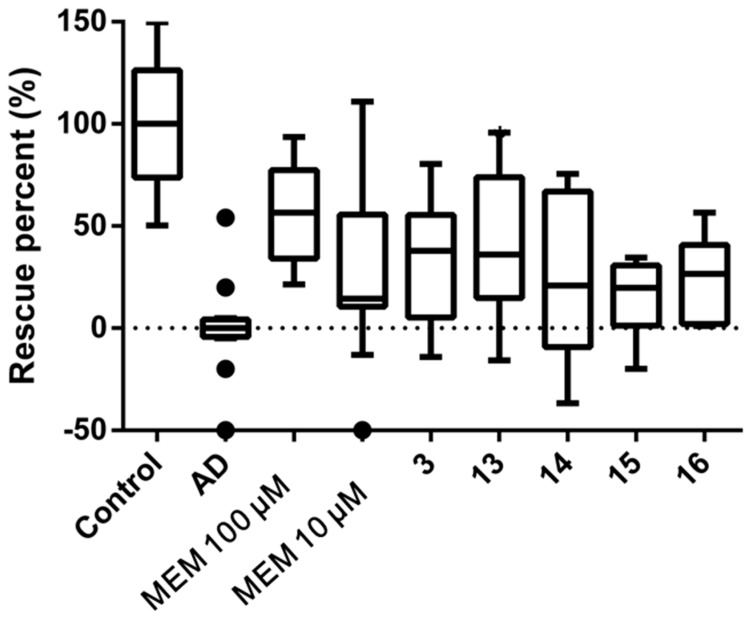
Differential effects of compound **3** and compounds **13**–**16** on rescuing memory deficit on a *Drosophila* AD model at 10 μM. The data are shown as means ± SD and analyzed by Tukey’s test; *n* ≥ 20.

The Pavlovian olfactory memory experiment was used to observe the rescue effect of compounds on memory loss. As analyzed from the DPPH and ThT assay results, compounds **3** and **13**–**16** were selected to explore whether they could rescue the memory loss of the *Drosophila* AD model. Compounds **3** and **13**–**16** were administered at a concentration of 10 µM. As shown in Figure 1, the PI value of the AD negative control group (P35*H29.3) was 27 (required less than 45). The PI difference between the genetic control group (P35*2U) and the AD negative group was 28 (required bigger than 15). Also, there was a significant difference between the memantine (MEM) 100 μM group and the AD group (*p* < 0.01). It demonstrated that the model flies had an obvious defect of learning and memory. In addition, the performance index (PI) value of compound **13** was 41.33, and there was a significant difference (*p* < 0.05) with the AD group. However, the PI values of compounds **3** and **14**–**16** are 39.66, 37.33, 33.16, and 36.83, respectively. It proved that gx-50 (compound **3**) and compounds **14**–**16** have a weak rescue effect on memory loss for AD flies. However, the rescue effect on memory loss of compound **13** was comparable to that of the positive control at the same concentration. Moreover, compound **13** was also far more effective than gx-50 (compound **3**). Overall, compound **13** should be a promising drug candidate for the treatment of AD. Figure 2 showed the standardization results for Figure 1.

In conclusion, twenty derivatives of gx-50 have been designed and synthesized. Among them, compound **13** showed the potent anti-oxidative activity, the inhibition and disaggregation on Cu^2+^-induced Aβ_1–42_ aggregation, and the rescue effect on memory loss of *Drosophila* AD model. Compared to gx-50 (compound **3**), compound **13** would be a much more potent anti-AD candidate for the development of relative drugs.

## 3. Experimental Section

### 3.1. General Experimental Procedures

Benzaldehyde, *p*-anisaldehyde, vanillin, and protocatechualdehyde were purchased from Sinopharm Chemical Reagent Co., Ltd. (Shanghai, China). Malonic acid was obtained from Xilong Chemical Co., Ltd. (Shantou, China). The compounds 4-methoxyphenethylamine, 2-phenylethylamine, 3,4-dimethoxyphenethylamine, and 3-methoxyphenethylamine were purchased from Energy Chemical (Shanghai, China). The compound 4-Dimethylaminopyridine was purchased from Macklin (Shanghai, China). The compound 1-(3-dimethylaminopropyl)-3-ethylcarbodiimide hydrochloride was purchased from Accela ChemBio Co. Ltd. (Shanghai, China). Column chromatography was performed with silica gel (200–300 mesh). All other reagents and solvents were analytical grade. NMR spectra were recorded by a Bruker AVANCE III-400 spectrometer (Bruker AXS GmbH, Karlsruhe, Germany) with tetramethylsilane (TMS) as an internal standard. The program MestReNova 9.0 was used to handle the NMR spectra.

### 3.2. General Procedure for the Preparation of Compounds ***1**–**20***

#### 3.2.1. Synthesis of Substituted Cinnamic Acids

The synthesis of substituted cinnamic acids was performed as Reference [30] described, with slight modifications. A mixture of substituted aromatic aldehydes (3 mmol), malonic acid (6 mmol), piperidine (0.3 mmol), was dissolved in pyridine and stirred at 90 °C for 4 h. Reaction progress was monitored by thin layer chromatography (TLC) analysis. When the reaction terminated, the pyridine was removed under vacuum. Then the reaction mixture was poured into ice water and washed by 2N HCl. Finally the precipitate was filtered and washed with hexane three times. After being dried under vacuum, the products were obtained.

#### 3.2.2. Synthesis of Gx-50 Derivatives **1**–**20**

The synthesis of gx-50 derivatives **1**–**20** was performed as Reference [31] described. EDCI (0.325 g, 1.5 mmol) and DMAP (0.183 g, 1.5 mmol) were added to a solution of a substituted cinnamic acid (1 mmol) in 10 mL of DMF. The reaction solution was stirred in an ice-water bath. After 15 min, phenolic amine (1 mmol) was added and the solution was stirred at room temperature overnight. When the reaction was completed as indicated by TLC analysis, the solution was washed with water and brine. Then the organic phase was dried with anhydrous MgSO_4_. The solvent was then evaporated under vacuum. The crude product was purified by silica gel column chromatography to get the target compounds **1**–**20** with high yields (95.8%, 96.8%, 97.5%, 96.1%, 96.6%, 94.2%, 95.8%, 97.0%, 95.7%, 95.6%, 98.3%, 96.4%, 98.8%, 97.2%, 98.3%, 96.0%, 98.4%, 97.9%, 96.8%, 98.3%, respectively).

#### 3.2.3. Purity Check of Compounds **1**–**20** by HPLC/MS Analysis

The purities of gx-50 derivatives **1**–**20** were analyzed by an HPLC/MS system with UV detection at 205 and 254 nm. Based on the integrity of the peak area, the purity of all compounds were determined over 97%. The HPLC analysis was performed on an Agilent 1200 Infinity system with an INNO C18 column (5 μm, 120 Å, 4.6 × 150 mm, Agilent Technologies, Santa Clara, CA, USA). The temperature of column chamber was kept at 25 °C, and the mobile phase at 1.0 mL/min was as follows: ACN in H_2_O containing 0.1% HCO_2_H (0–30 min: from 0% to 100%).

Compound **12**: Colorless amorphous powder; ^1^H-NMR (400 MHz, CDCl_3_) *δ* 7.53 (d, *J* = 15.2 Hz, 1H), 7.23 (m, 1H), 7.02 (m, 1H), 6.95 (m, 1H), 6.89 (d, *J* = 8.0 Hz, 1H), 6.79 (m, 3H), 6.19 (d, *J* = 15.6 Hz, 1H), 5.74 (s, 1H), 3.87 (s, 3H), 3.78 (s, 3H), 3.65 (m, 2H), 2.86 (t, *J* = 6.8 Hz, 2H). ^13^C-NMR (101 MHz, CDCl_3_) *δ* 161.50, 155.10, 142.71, 142.02, 136.35, 135.80, 124.93, 122.55, 117.38, 116.38, 113.38, 110.01, 109.76, 107.14, 104.91, 51.17, 50.46, 35.91, 30.98. ESI/MS calculated for C_19_H_21_NNaO_4_: 350.1363, found: 350.1365 [M + H]^+^.

Compound **16**: yellow amorphous powder; ^1^H-NMR (400 MHz, DMSO-*d_6_*) *δ* 9.34 (s, 1H), 9.10 (s, 1H), 8.04 (t, *J* = 5.2 Hz, 1H), 7.21 (m, 2H), 6.93 (s, 1H), 6.77 (m, 5H), 6.32 (d, *J* = 15.6 Hz, 1H), 3.73 (s, 3H), 3.38 (m, 2H), 2.74 (t, *J* = 7.2 Hz, 2H). ^13^C-NMR (101 MHz, DMSO-*d_6_*) *δ* 170.55, 164.48, 152.47, 150.73, 146.33, 144.21, 134.53, 131.59, 126.09, 125.58, 123.71, 120.94, 119.44, 118.99, 116.79, 60.10, 40.45, 35.89. ESI/MS calculated for C_18_H_19_NNaO_4_: 336.1206, found: 336.1201 [M + Na]^+^.

Compound **20**: Colorless amorphous powder; ^1^H-NMR (400 MHz, CDCl_3_) *δ* 7.53 (d, *J* = 15.0 Hz, 1H), 7.23 (m, 1H), 6.97 (m, 2H), 6.80 (m, 4H), 6.14 (d, *J* = 15.6 Hz, 1H), 5.98 (s, 2H), 5.55 (s, 1H), 3.80 (s, 3H), 3.65 (q, *J* = 6.6 Hz, 2H), 2.86 (t, *J* = 6.8 Hz, 2H). ^13^C-NMR (101 MHz, CDCl_3_) *δ* 165.96, 159.87, 149.05, 148.22, 140.81, 140.52, 129.70, 129.21, 123.83, 121.11, 118.59, 114.49, 111.93, 108.52, 106.35, 101.43, 55.21, 40.65, 35.73. ESI/MS calculated for C_19_H_19_NNaO_4_: 348.1206, found: 348.1204 [M + Na]^+^.

### 3.3. Biological Assay

#### 3.3.1. DPPH Assay

DPPH was dissolved in ethanol (0.1 mM) daily and stored in the dark. Before usage, its absorbance was calibrated to 0.7 using ethanol by a microplate reader. Ethanol was used as a negative control and Vitamin C (Vc) was used as a positive control. A series of sample solutions with different concentrations in DMSO were prepared, and then 2 μL of each sample solution was removed and added into 96-well plates respectively. Then 198 μL of DPPH solution was added, and each group had 3 parallel holes. The blank group and the positive control group had the same treatment as the sample group. The 96-well plate was shaken in the SPH-2000 shaking incubator (Xi’an Heb Biotechnology Co., Ltd., Shanghai, China) at 37 °C for 30 min in the dark. Consequently, the absorbance was measured at 517 nm with a RT-6100 microplate reader (Rayto, Shenzhen, China). The inhibition percent of each test compound was calculated based on the equation: DPPH inhibition percent (%) = [(A_C_ − A_S_)/A_C_] × 100, where Ac represents the absorbance of the control sample and As is the absorbance of the test compound.

#### 3.3.2. ThT Assay

The dried Aβ_1–42_ peptide powder (purity ≥ 95%, Aladdin, Shanghai, China) was dissolved in 1,1,1,3,3,3-hexafluoro-2-propanol (HFIP) at a concentration of 1.0 mg·mL^−1^. The HFIP/peptide solution was shaken at room temperature for 6 h to gain the monomeric form of Aβ_1–42_. Then, the HFIP/peptide solution was dried under a gentle stream of nitrogen gas for 2 h. The dried peptide power was then dissolved with anhydrous DMSO and stored at −20 °C. For the test of copper-mediated Aβ_1–42_ inhibition and disaggregation, the procedures were carried out according to the literature [32]. In brief, the above Aβ_1–42_ stock solution was diluted in 20 μM HEPES (pH 6.6) with 150 μM NaCl to the desired final concentration before use. Either 10 μL of 25 μM peptide solution and 10 μL of test compound solution were incubated at 37 °C for 24 h together or the addition of peptide solution and compound solution was accomplished by two steps in an interval of 24 h, it was incubated at 37 °C for 24 h continuously. Then 180 μL of 50 mM glycine-NaOH buffer (pH 8.0) containing thioflavin T (5 μM) was added to dilute the above sample solution. After 5 min, the fluorescence intensities were measured (excitation, 450 nm; emission, 485 nm) by a Horiba FluoroMax4 spectrophotometer (HORIBA Scientific, Edison, NJ, USA). The percent inhibition of aggregation was calculated via the expression (1 − IF_i_/IF_c_) × 100%, where IF_i_ and IF_c_ were the fluorescence intensities for Aβ_1–42_ in the presence and absence of test compounds after subtracting the background, respectively.

### 3.4. Pavlov’s Olfactory Memory Test

#### 3.4.1. Fly Stocks and Culture

All flies used in this study have been backcrossed for at least five generations, which could make them maintain the genetic background of w^1118^(isoCJ1). Therefore, the strain of w^1118^ (isoCJ1) (“2U”) was an isogenic line used as a control in the experiments. In order to eliminate the potential adverse effects of long-term expression of Aβ42 protein on *Drosophila* strains, only when the flies (“H29.3”) that carried the human Aβ42 gene crossed with flies of elav-GAL4^c155^ (“P35”) that could start the expression of Aβ42 gene, its offspring could express Aβ42 protein in the nervous system [33,34,35]. However, offspring flies obtained by crossing P35 and 2U could not express Aβ42 protein. The learning test was conducted using the above flies. All flies were maintained on a 12 h light per day (light from 07:00 to 19:00) at 24 °C and 40–60% relative humidity. Control male flies (elav/Y; +/+) and experimental male flies (elav/Y; UAS-Aβ42/+) were selected by microscope on the second day after eclosion. The selected flies were reared at 24 °C and 40 ± 15% relative humidity with standard corn meal food for subsequent drug treatment.

#### 3.4.2. Drug Intervention

All compounds and memantine (purity ≥ 98%, Aladdin, Shanghai, China) were dissolved in a vehicle solution, which was composed of 4% sucrose and 1% DMSO. Subsequently, the final concentration of each compound was diluted to 10 μM with 4% sucrose solution. From the second day to eighth day after eclosion, each group of flies were continuously administered 4 hours per day and then rested in a fresh food environment for 20 h. For the test compound group, flies were fed with 50 μL of compound solution daily, while control group of flies were fed with the same amount of sugar water. In this experiment, six data points were measured for each group flies, and each final data was the average of olfactory memory of two groups [36,37,38].

#### 3.4.3. *Drosophila* Olfactory Escape Behavior Test

The task was performed according to a previous reported method with minor modifications [36,37,38]. Learning and memory tests were conducted at 25 °C and 70% relative humidity in the dark. During the training period, about one hundred flies were loaded into a copper-lined training tube. These flies were successively exposed to two odors, 3-octanol (OCT) and 4-methylcyclohexanol (MCH) for 60 s. Then the fresh air was ventilated for 45 s. Flies would receive an electric shock for 1 minute when they smelt the first odor. To avoid the natural preference of flies for odors, one tube of flies would smell the OCT firstly and receive an electric shock, while the other tube would smell the MCH firstly and receive an electric shock at each data point. The experimental average value of two tubes of the flies was considered as a data point. Then flies were immediately transferred to the choice point of a T-maze and forced to select between the OCT and MCH containers for two minutes. Consequently, the flies in different containers were counted and a performance index (PI) was calculated. If the flies had a 50:50 distribution in the T-maze test, it means the PI = 0 and the flies could not remember the connection of the electric shock and the odor. Additionally, the PI = 100 means that 100% of flies could remember the connection between the odor and the electric shock.

## 4. Statistical Analysis

All data were expressed as mean ± standard deviation (SD), and results were subjected to Tukey’s test or one-way analysis of variance (ANOVA). *p* < 0.05 was accepted to indicate the significance.

## Figures and Tables

**Table 1:** The DPPH assay result for compounds **1**–**20**.

Compound	R_1_	R_2_	R_3_	R_4_	Inhibition Ratio (%)		
					50 μM	100 μM	200 μM
**1**	H	H	OCH_3_	H	2.97 ± 1.67	4.35 ± 2.29	4.93 ± 2.13
**2**	H	H	H	H	3.1 ± 0.30	6.69 ± 0.37	9.20 ± 0.67
**3**	H	H	OCH_3_	OCH_3_	2.92 ± 1.7	5.03 ± 2.01	6.18 ± 0.65
**4**	H	H	H	OCH_3_	0.99 ± 0.55	2.91 ± 1.24	3.13 ± 0.59
**5**	H	OCH_3_	OCH_3_	H	4.78 ± 2.8	9.78 ± 0.61	11.22 ± 0.43
**6**	H	OCH_3_	H	H	1.08 ± 0.70	5.11 ± 2.06	12.91 ± 1.57
**7**	H	OCH_3_	OCH_3_	OCH_3_	3.61 ± 0.89	5.58 ± 0.78	9.58 ± 1.39
**8**	H	OCH_3_	H	OCH_3_	0.06 ± 2.63	2.81 ± 2.78	3.42 ± 0.78
**9**	OCH_3_	OH	OCH_3_	H	30.21 ± 1.52	36.28 ± 3.43	46.57 ± 0.86
**10**	OCH_3_	OH	H	H	16.69 ± 0.46	34.06 ± 2.57	44.07 ± 2.20
**11**	OCH_3_	OH	OCH_3_	OCH_3_	17.43 ± 0.46	40.28 ± 0.21	50.19 ± 1.90
**12**	OCH_3_	OH	H	OCH_3_	28.74 ± 0.78	46.93 ± 0.42	51.83 ± 1.42
**13**	OH	OH	OCH_3_	H	54.34 ± 1.44	56.16 ± 1.78	56.37 ± 2.86
**14**	OH	OH	H	H	54.21 ± 1.21	55.01 ± 0.35	54.17 ± 2.62
**15**	OH	OH	OCH_3_	OCH_3_	57.85 ±0.15	59.96 ± 0.79	58.33 ± 2.71
**16**	OH	OH	H	OCH_3_	58.86 ±0.43	60.85 ± 0.37	57.68 ± 0.54
**17**	OCH_3_	H	-	-	2.13 ± 0.32	4.60 ± 0.33	4.82 ± 0.36
**18**	H	H	-	-	0.63 ± 0.27	1.99 ± 1.50	2.27 ± 1.52
**19**	OCH_3_	OCH_3_	-	-	1.44 ± 1.68	1.39 ± 0.85	2.02 ± 0.52
**20**	H	OCH_3_	-	-	1.57 ± 0.13	4.09 ± 0.31	4.53 ± 0.83
Vc					41.42 ± 2.83	72.00 ± 1.15	78.88 ± 0.80

Values are expressed as the means ± SD of at least three independent experiments.

**Table 2:** Thioflavin T (ThT) assay results for compounds **1**–**20**.

Compound	R_1_	R_2_	R_3_	R_4_	Aβ Aggregation (inhib. %) ^a^	Disaggregation (%) ^b^
**1**	H	H	OCH_3_	H	4.83 ± 8.10	4.40 ± 2.11
**2**	H	H	H	H	0.14 ± 1.49	0.27 ± 0.94
**3**	H	H	OCH_3_	OCH_3_	1.73 ± 2.15	2.39 ± 1.35
**4**	H	H	H	OCH_3_	2.28 ± 3.24	3.85 ± 2.22
**5**	H	OCH_3_	OCH_3_	H	14.47 ± 4.15	11.92 ± 2.32
**6**	H	OCH_3_	H	H	12.26 ± 7.37	11.19 ± 1.70
**7**	H	OCH_3_	OCH_3_	OCH_3_	7.87 ± 2.90	9.04 ± 1.99
**8**	H	OCH_3_	H	OCH_3_	3.33 ± 1.26	4.49 ± 0.49
**9**	OCH_3_	OH	OCH_3_	H	2.72 ± 3.65	1.05 ± 1.04
**10**	OCH_3_	OH	H	H	5.94 ± 3.13	4.16 ± 1.65
**11**	OCH_3_	OH	OCH_3_	OCH_3_	4.37 ± 3.20	2.04 ± 0.71
**12**	OCH_3_	OH	H	OCH_3_	2.78 ± 4.27	0.33 ± 0.45
**13**	OH	OH	OCH_3_	H	56.43 ± 1.72	52.76 ± 0.33
**14**	OH	OH	H	H	60.68 ± 1.76	57.28 ± 3.61
**15**	OH	OH	OCH_3_	OCH_3_	61.85 ± 1.70	64.44 ± 0.76
**16**	OH	OH	H	OCH_3_	38.47 ± 2.37	51.51 ± 1.42
**17**	OCH_3_	H	−	−	14.34 ± 2.37	15.50 ± 0.07
**18**	H	H	−	−	3.47 ± 7.57	7.74 ± 2.00
**19**	OCH_3_	OCH_3_	−	−	13.06 ± 4.58	18.68 ± 1.17
**20**	H	OCH_3_	−	−	12.93 ± 3.66	0.61 ± 0.38
curcumin					48.85 ± 1.20	43.83 ± 1.22
resveratrol					60.49 ± 2.71	51.27 ± 3.31

^a^ The inhibition percentage Cu^2+^-induced Aβ_1–42_ aggregation. ^b^ The disaggregation percentage of Cu^2+^-induced Aβ_1–42_ aggregates. Assays were carried out in the presence of 25 μM inhibitor and 25 μM A*β*_1–42_. Values are expressed as the means ± SD of at least three independent experiments.
